# Supplementary material for: Assessment of Bidirectional Relationships between Leisure Sedentary Behaviors and Neuropsychiatric Disorders: A Two-Sample Mendelian Randomization Study
Source: Genes (Basel). 2022 May 27;13(6):962. doi: 10.3390/genes13060962 (PMC9223103; doi:10.3390/genes13060962)
Supplement: Supplementary file 1 [file genes-13-00962-s001.zip › Supplementary Figures S1-S12.pdf]

Supplementary Figures

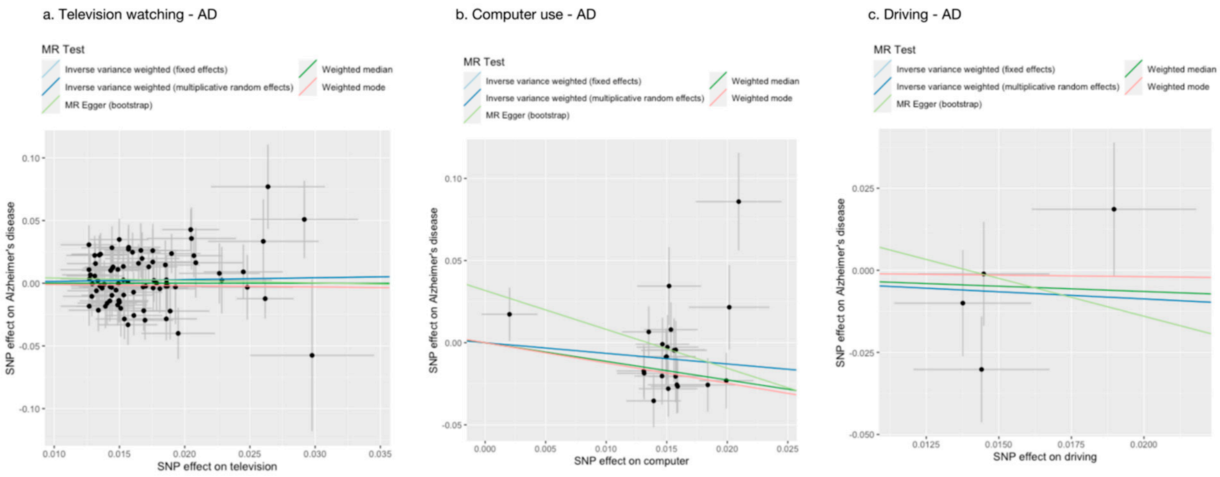

Supplementary Figure S1. Scatter plots of leisure sedentary behaviors and Alzheimer's disease (AD).

Notes: Scatter plot including the MR estimates between leisure sedentary behaviors and Alzheimer's disease (AD), in which a threshold of $P < 1 \times 10^{-8}$ was used for the selection of variants. The variants' effect on leisure sedentary behaviors are displayed on X-axis, the variants' effect on Alzheimer's disease (AD) are displayed on the Y-axis. a. Scatter plot of SNP effects on television watching versus their effects on Alzheimer's disease (AD). b. Scatter plot of SNP effects on computer use versus their effects on Alzheimer's disease (AD). c. Scatter plot of SNP effects on driving versus their effects on Alzheimer's disease (AD). SNP = single nucleotide polymorphism, MR = Mendelian randomization.

a. Television watching - AD

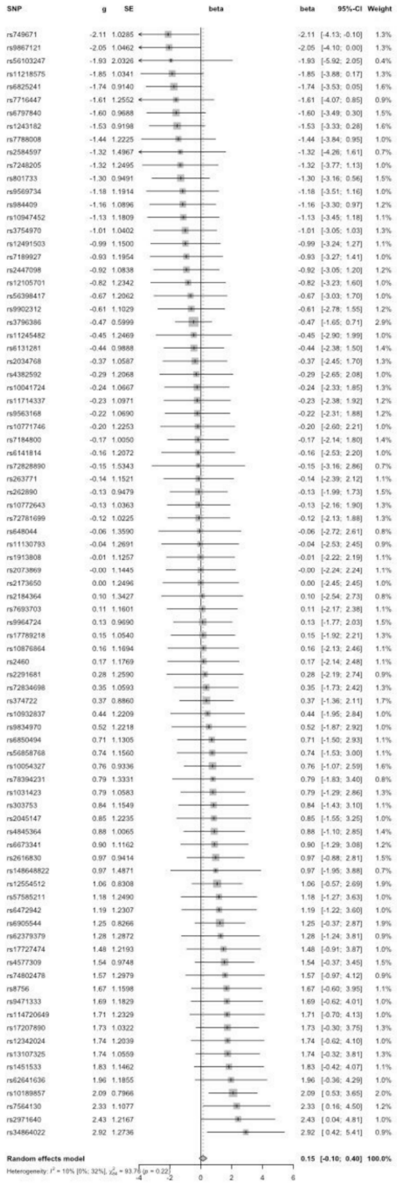

b. Computer use - AD

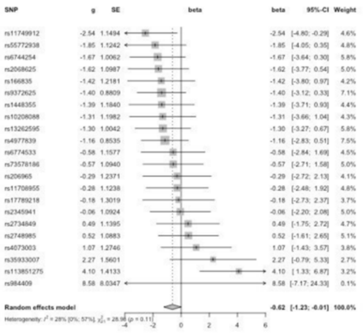

c. Driving - AD

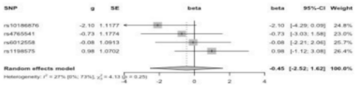

Supplementary Figure S2. Forest plots of leisure sedentary behaviors genetic variants associations with Alzheimer's disease (AD).

Notes: The Mendelian randomization effect size of leisure sedentary behaviors product are displayed on the X-axis. The different genetic variants for leisure sedentary behaviors are listed on the Y-axis. a. Forest plot of television watching genetic variants association with AD. b. Forest plot of computer use genetic variants association with Alzheimer's disease (AD). Forest plot of driving genetic variants association with AD.

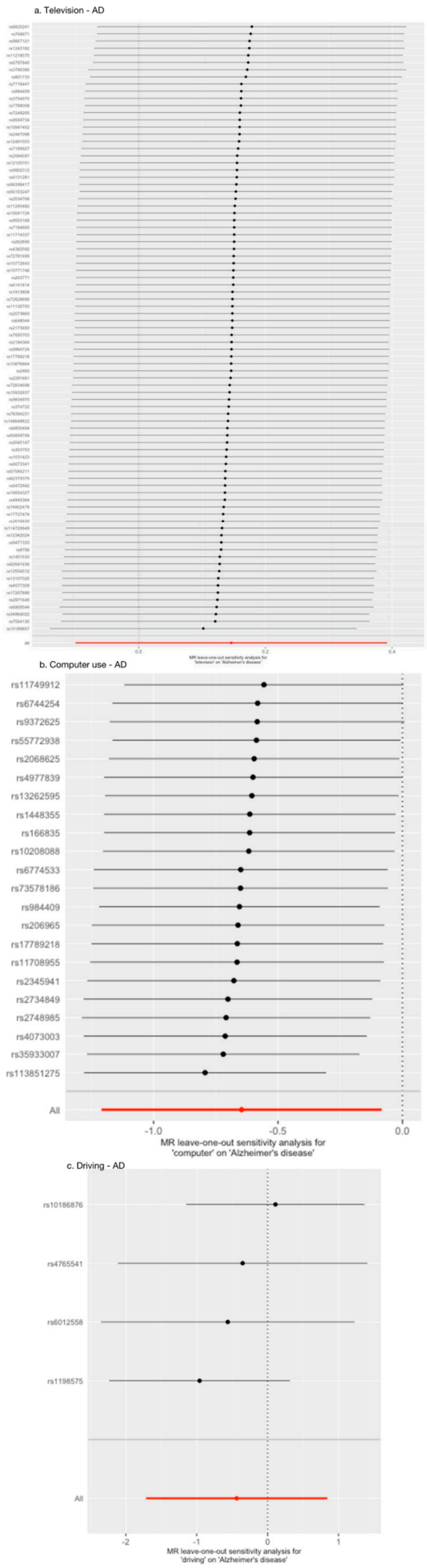
a. Television - AD
All
0.0
0.2
0.4
b. Computer use - AD
rs11749912
rs6744254
rs9372625
rs55772938
rs2068625
rs4977839
rs13262595
rs1448355
rs166835
rs10208088
rs6774533
rs73578186
rs984409
rs206965
rs17789218
rs11708955
rs2345941
rs2734849
rs2748985
rs4073003
rs35933007
rs113851275
All
-1.0
-0.5
0.0
MR leave-one-out sensitivity analysis for
'computer' on 'Alzheimer's disease'
c. Driving - AD
rs10186876
rs4765541
rs6012558
rs1198575
All
-2
-1
0
1
MR leave-one-out sensitivity analysis for
'driving' on 'Alzheimer's disease'

Supplementary Figure S3. Leave-One-Out analyses for variants associated with leisure sedentary behaviors on Alzheimer's disease (AD).

Notes: The influence of single variant on the overall MR analysis of AD was indicated by sequential removal of each genetic variant in the television watching (a), computer use (b) and driving (c). MR = Mendelian randomization.

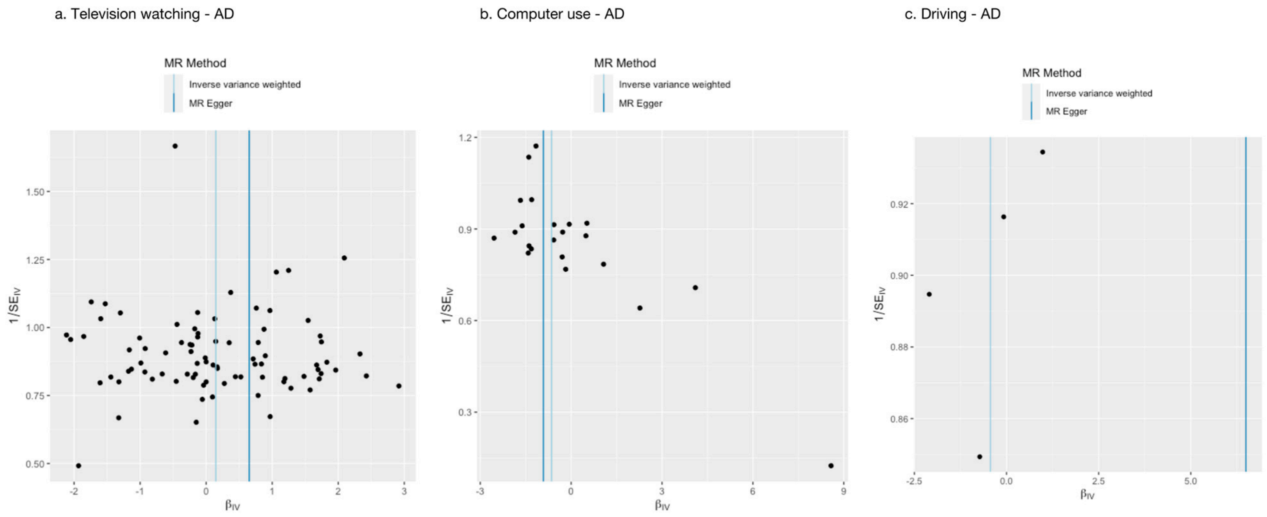

Supplementary Figure S4. Funnel plots for the effects of leisure sedentary behaviors on Alzheimer's disease (AD).

Notes: The causal effect of individual variant against the overall estimate in the MR analysis on television watching (a), computer use (b), driving (c) and Alzheimer's disease (AD) was depicted. Vertical lines denote overall estimates by the inverse variance weighted method. IV = instrumental variable; SE = standard error.

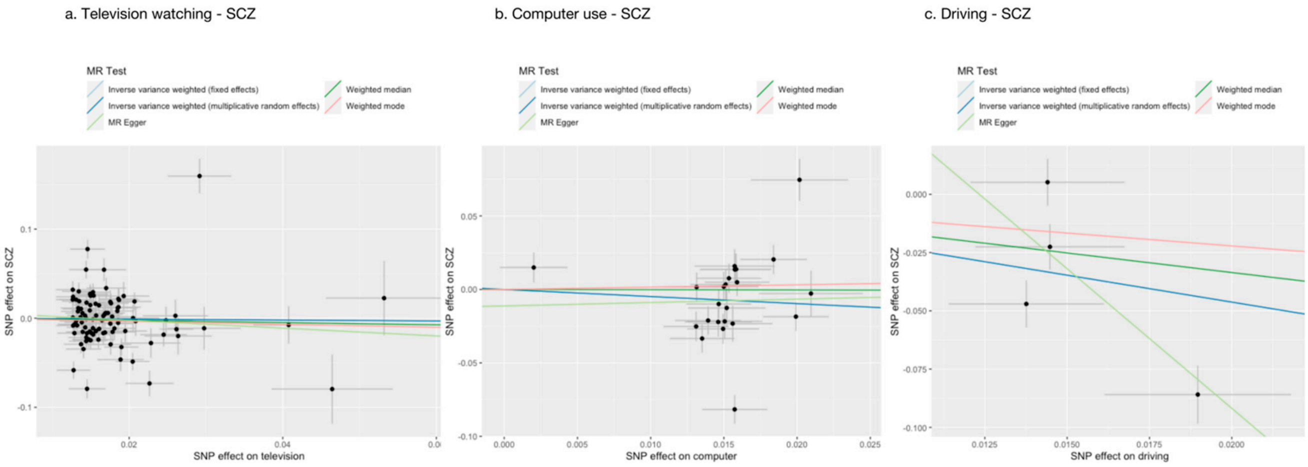

Supplementary Figure S5. Scatter plots of leisure sedentary behaviors and Schizophrenia (SCZ).

Notes: Scatter plot including the MR estimates between leisure sedentary behaviors and Schizophrenia (SCZ), in which a threshold of $P < 1 \times 10^{-8}$ was used for the selection of variants. The variants' effect on leisure sedentary behaviors are displayed on X-axis, the variants' effect on Schizophrenia (SCZ) are displayed on the Y-axis. a. Scatter plot of SNP effects on television watching versus their effects on Schizophrenia (SCZ). b. Scatter plot of SNP effects on computer use versus their effects on Schizophrenia (SCZ). c. Scatter plot of SNP effects on driving versus their effects on Schizophrenia (SCZ). SNP = single nucleotide polymorphism, MR = mendelian randomization.

a. Television watching - SCZ

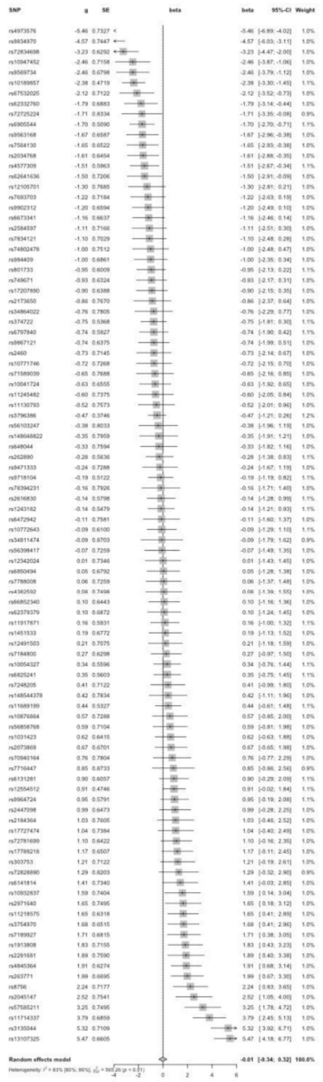

b. Computer use - SCZ

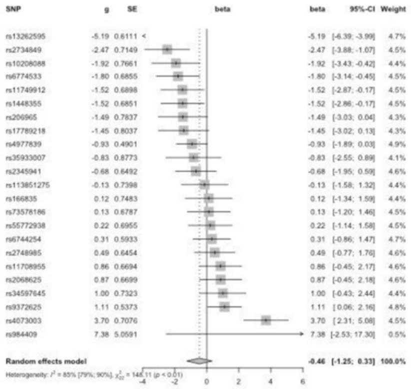

c. Driving - SCZ

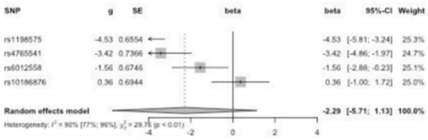

Supplementary Figure S6. Forest plots of leisure sedentary behaviors genetic variants associations with Schizophrenia (SCZ).

Notes: The Mendelian randomization effect size of leisure sedentary behaviors product are displayed on the X-axis. The different genetic variants for leisure sedentary behaviors are listed on the Y-axis. a. Forest plot of television watching genetic variants association with Schizophrenia (SCZ). b. Forest plot of computer use genetic variants association with Schizophrenia (SCZ). Forest plot of driving genetic variants association with SCZ.

a. Television - SCZ

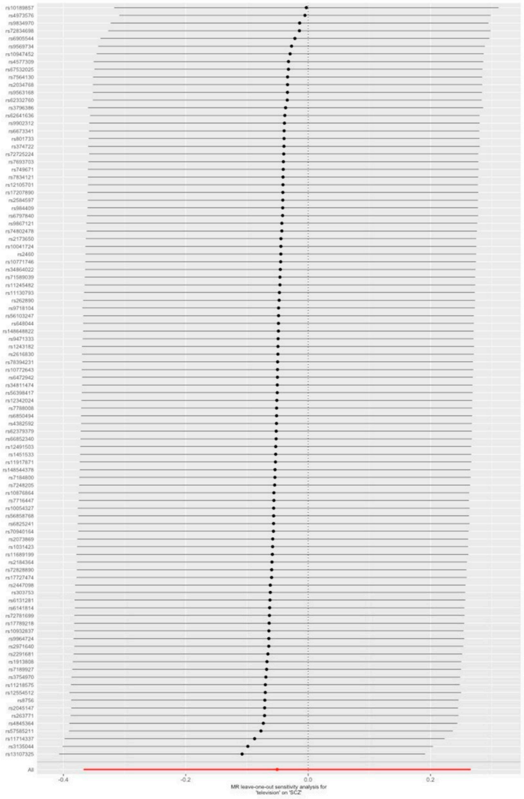

b. Computer use - SCZ

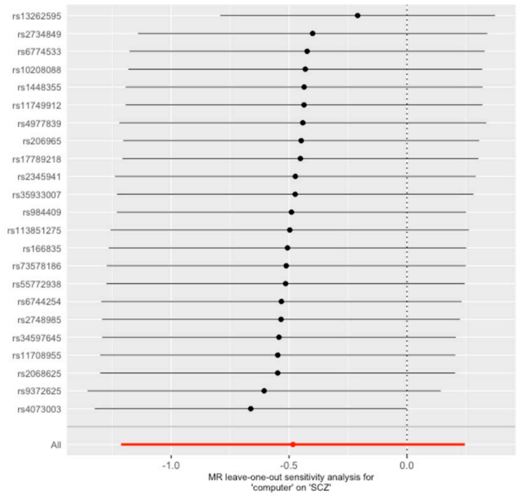

c. Driving - SCZ

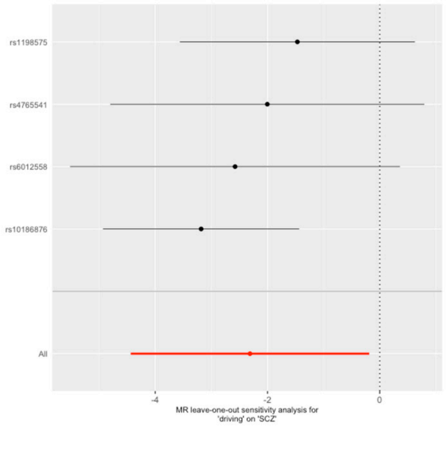

Supplementary Figure S7. Leave-One-Out analyses for variants associated with leisure sedentary behaviors on Schizophrenia (SCZ).

Notes: The influence of single variant on the overall MR analysis of Schizophrenia (SCZ)was indicated by sequential removal of each genetic variant in the television watching (a), computer use (b) and driving (c). MR = Mendelian randomization.

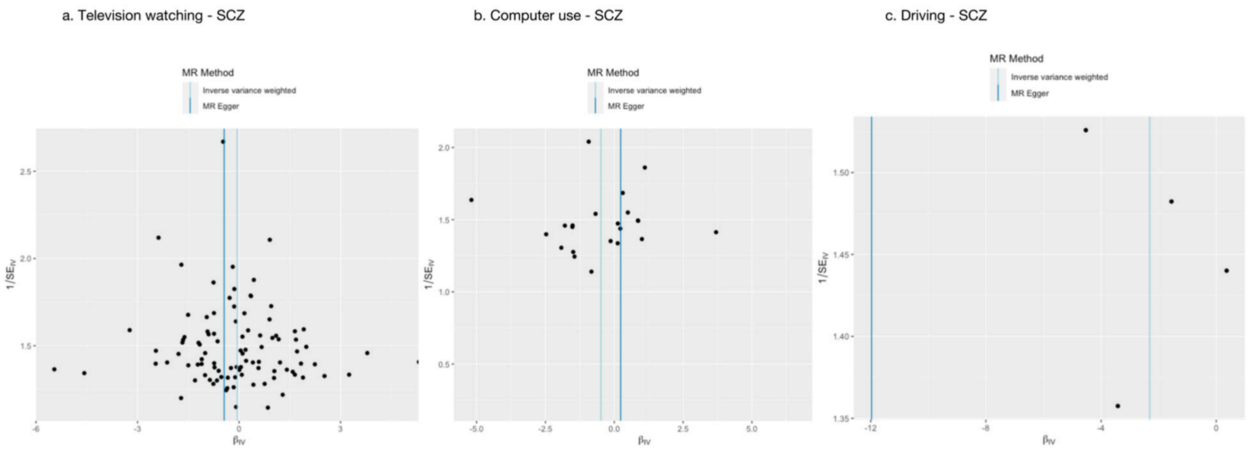

Supplementary Figure S8. Funnel plots for the effects of leisure sedentary behaviors on Schizophrenia (SCZ).

Notes: The causal effect of individual variant against the overall estimate in the MR analysis on television watching (a), computer use (b), driving (c) and Schizophrenia (SCZ) was depicted. Vertical lines denote overall estimates by the inverse variance weighted method. IV = instrumental variable; SE = standard error.

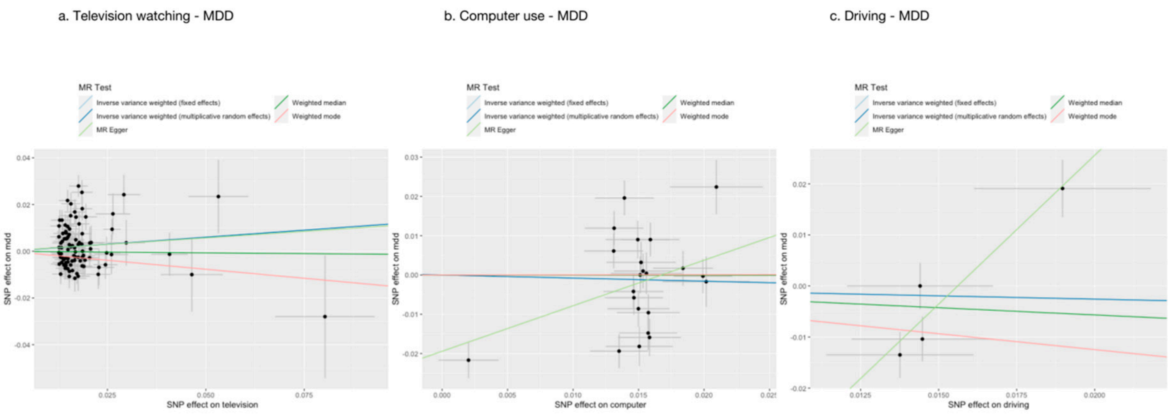

Supplementary Figure S9. Scatter plots of leisure sedentary behaviors and Major depression disorder (MDD).

Notes: Scatter plot including the MR estimates between leisure sedentary behaviors and Major depression disorder (MDD), in which a threshold of $P < 1 \times 10^{-8}$ was used for the selection of variants. The variants' effect on leisure sedentary behaviors are displayed on X-axis, the variants' effect on Major depression disorder (MDD) are displayed on the Y-axis. a. Scatter plot of SNP effects on television watching versus their effects on Major depression disorder (MDD). b. Scatter plot of SNP effects on computer use versus their effects on Major depression disorder (MDD). c. Scatter plot of SNP effects on driving versus their effects on Major depression disorder (MDD). SNP = single nucleotide polymorphism, MR = Mendelian randomization.

a. Television watching - MDD

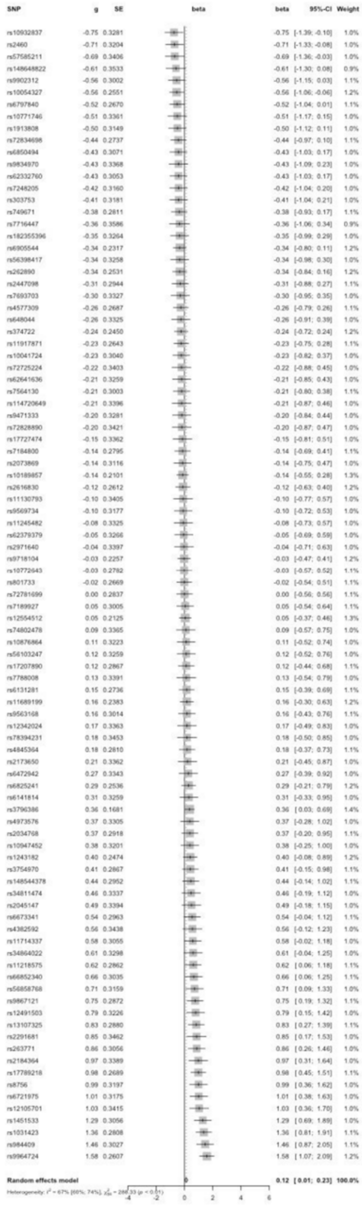

b. Computer use - MDD

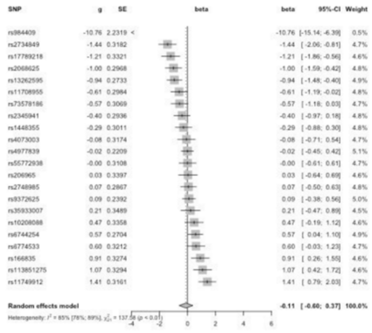

c. Driving - MDD

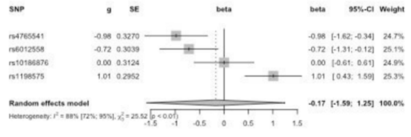

Supplementary Figure S10. Forest plots of leisure sedentary behaviors genetic variants associations with Major depression disorder (MDD).

Notes: The Mendelian randomization effect size of leisure sedentary behaviors product are displayed on the X-axis. The different genetic variants for leisure sedentary behaviors are listed on the Y-axis. a. Forest plot of television watching genetic variants association with Major depression disorder (MDD). b. Forest plot of computer use genetic variants association with Major depression disorder (MDD). Forest plot of driving genetic variants association with Major depression disorder (MDD).

a. Television - MDD

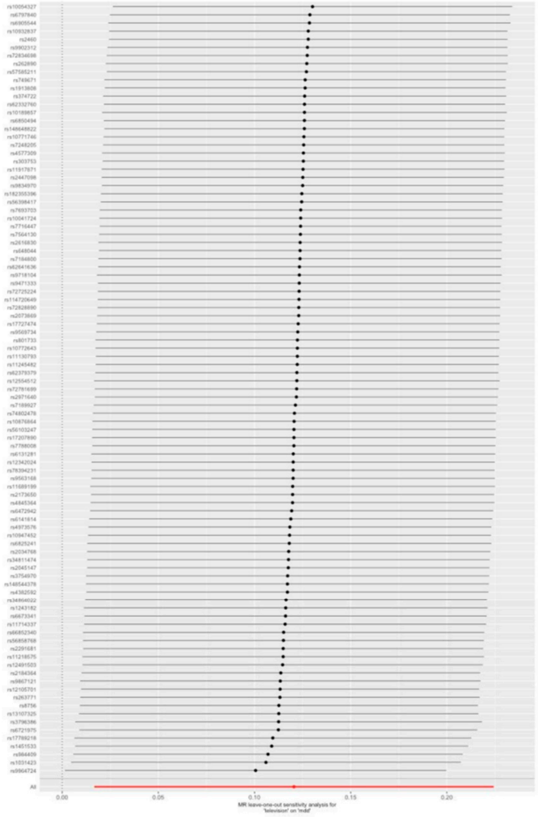

b. Computer use - MDD

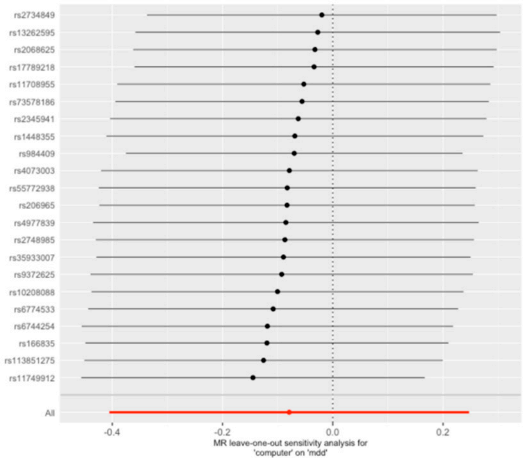

c. Driving - MDD

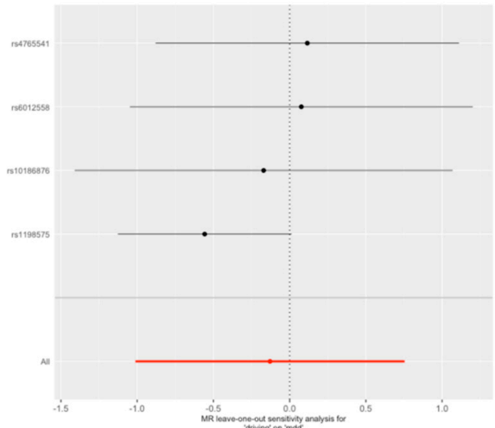

Supplementary Figure S11. Leave-One-Out analyses for variants associated with leisure sedentary behaviors on Major depression disorder (MDD).

Notes: The influence of single variant on the overall MR analysis of Major depression disorder (MDD) was indicated by sequential removal of each genetic variant in the television watching (a), computer use (b) and driving (c). MR = Mendelian randomization.

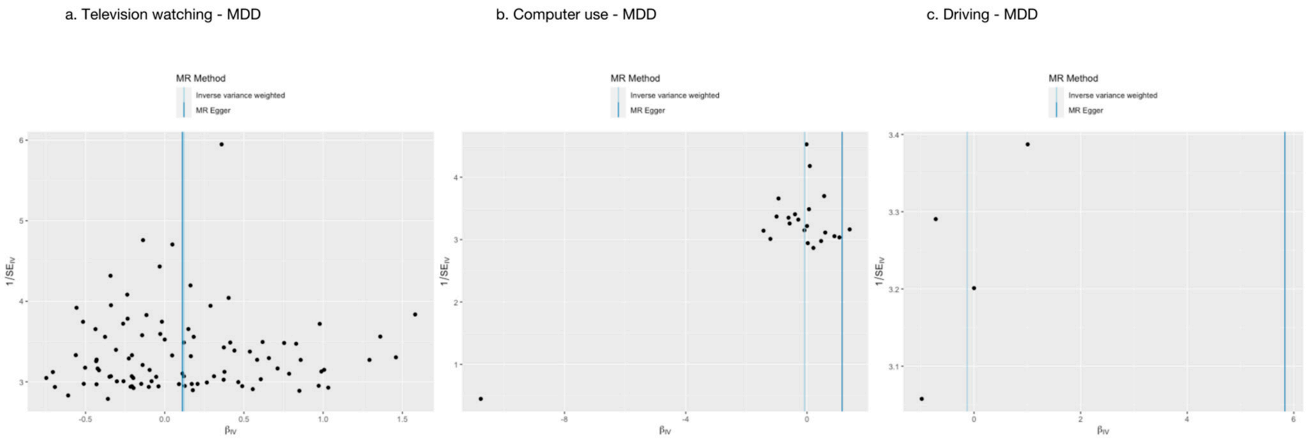

Supplementary Figure S12. Funnel plots for the effects of leisure sedentary behaviors on Major depression disorder (MDD).

Notes: The causal effect of individual variant against the overall estimate in the MR analysis on television watching (a), computer use (b), driving (c) and Major depression disorder (MDD) was depicted. Vertical lines denote overall estimates by the inverse variance weighted method. IV = instrumental variable; SE = standard error.
